# Clinical and biological factors associated with response to immune checkpoint inhibitors in advanced sarcomas: IMPRESARC, a French retrospective multicenter cohort study

**DOI:** 10.1002/cncr.70052

**Published:** 2025-09-05

**Authors:** Mina Fazel, Romain Varnier, Hélène Vanacker, Nicolas Penel, Sarah Watson, Benjamin Verret, Thibaud Valentin, Emmanuelle Bompas, Waisse Waissi, Alexandra Meurgey, Françoise Ducimetière, Jean‐Yves Blay, Armelle Dufresne, Mehdi Brahmi

**Affiliations:** ^1^ Department of Medical Oncology Centre Léon Bérard Lyon France; ^2^ Faculté de Médecine Lyon Est Université Claude Bernard Lyon Lyon France; ^3^ Department of Medical Oncology Centre Oscar‐Lambret Lille France; ^4^ ULR 2694‐Metrics: Évaluation des Technologies de Santé et des Pratiques Médicales CHU de Lille University of Lille Lille France; ^5^ Department of Medical Oncology Institut Curie Paris France; ^6^ INSERM U830 Institut Curie Paris France; ^7^ Department of Medical Oncology Institut Gustave Roussy Villejuif France; ^8^ INSERM U981 Institut Gustave Roussy Villejuif France; ^9^ Department of Medical Oncology IUCT‐Oncopole Toulouse France; ^10^ Department of Medical Oncology Institut de Cancérologie de l'Ouest Nantes France; ^11^ NETSARC+ Transversal Task Force Lyon France

**Keywords:** immune checkpoint inhibitor, immunotherapy, predictive factors, sarcoma

## Abstract

**Background:**

Immune checkpoint inhibitors (ICIs) in unselected sarcomas yield limited response rates and tumor control. Long‐term responders have however been reported, suggesting a critical challenge in refining patient selection, by identifying reliable predictive factors for response.

**Methods:**

The authors conducted a multicenter, retrospective study of patients with advanced sarcomas treated with ICIs in six French reference sarcoma centers. The study assessed efficacy and safety, as well as clinical and biological variables associated with objective response rate (ORR), progression‐free survival (PFS), and overall survival (OS).

**Results:**

A total of 272 patients were included in the analysis. The ORR was 17%, with 16% partial responses and 1% complete responses. Stable disease (SD) occurred in 33% of patients, resulting in a disease control rate of 49%. Median PFS was 2.7 months (95% confidence interval [CI], 2.5–3.4), with 28% of patients showing PFS >6 months and 13% with PFS >12 months. Median OS was 13.5 months (95% CI, 11.0–17.3). The safety profile was consistent with that of clinical trials, with 5% of patients experiencing grade ≥3 adverse events and 10% discontinuing treatment due to toxicity. Poorer outcomes were associated with high Eastern Cooperative Oncology Group performance status (≥1), cyclophosphamide coadministration, and high derived neutrophil‐to‐lymphocyte ratio. Anti‐programmed death‐ligand 1 (PD‐L1) therapies were associated with shorter OS compared to anti–PD‐1. Histotypes such as alveolar soft part sarcoma (ASPS) had better survival, whereas dedifferentiated liposarcoma had poorer outcomes.

**Conclusions:**

Despite a short median PFS, certain histological subtypes, including ASPS, chordomas, SMARCA4‐deficient tumors, gastro‐intestinal stromal tumor, and *NF1* mutations, showed strong activity signals, indicating long‐term responses in some patients.

## Abstract 2

This study offers one of the largest multicentric analyses of advanced sarcoma patients treated with immune checkpoint inhibitors (ICIs), highlighting the predictive value of histological subtype and identifying potential roles for specific molecular alterations, such as *NF1*. The presence of long‐term responders underscores the therapeutic potential of ICIs in selected sarcoma subsets, offering new insights to guide personalized treatment strategies.

## INTRODUCTION

Sarcomas gather a heterogeneous group of rare malignant tumors originating from mesenchymal tissues, representing 1% of all adult malignant tumors.^1^, ^2^ They can occur at any site and any age and are traditionally classified into soft tissue sarcomas (STS) and bone sarcomas (BS). Significant heterogeneity persists within these subgroups, with over 150 distinct subtypes identified in the 5th World Health Organization Classification of Soft Tissue and Bone Tumors.^2^, ^3^ Although complete en bloc R0 surgery serves as the mainstay of localized sarcoma management, treating advanced sarcomas remains challenging due to their rarity and diversity.^4^, ^5^ Patients are typically treated with conventional cytotoxic chemotherapy and antiangiogenic tyrosine kinase inhibitors (TKI), yielding median progression‐free survival (PFS) of 3–6 months as first‐line treatment and median overall survival (OS) of less than 2 years.^6^, ^7^, ^8^, ^9^, ^10^, ^11^, ^12^, ^13^ Consequently, the management of advanced sarcomas represents a significant unmet medical need.

Over the past decade, modern immunotherapy has witnessed substantial growth in the treatment landscape of numerous solid and hematologic cancers.^14^ However, early experiences with immunotherapy in trials involving the use of immune checkpoint inhibitors (ICIs) in unselected sarcomas have yielded disappointing outcomes,^15^, ^16^, ^17^, ^18^, ^19^, ^20^, ^21^ with response rates ranging from 0% to 20% and a median PFS of 2–3.5 months. A specific histological subtype, alveolar soft part sarcoma (ASPS), displays higher response rates and extended PFS,^16^, ^17^, ^20^, ^21^, ^22^ leading to a recent Food and Drug Administration approval of atezolizumab, an anti–programmed death‐ligand 1 (PD‐L1) antibody. In chordomas, a promising signal of efficacy was reported in case series.^23^ The efficacy in these two specific subtypes was further confirmed in Acsé Pembrolizumab basket trial.^24^ Recently, the presence of tertiary lymphoid structures (TLS), observed in nearly 20% of sarcomas, was identified as a predictive factor for anti–PD‐1/PD‐L1 efficacy.^25^ The theragnostic utility of this marker was further validated in a prospective study.^18^

Most studies evaluating the use of immunotherapy included only few patients with various histological subtypes, making it difficult to decipher predictive factors.

The objective of this study was to identify clinical and biological factors associated with ICI efficacy (PFS and OS).

## MATERIALS AND METHODS

### Study population

All data from adult patients diagnosed with an unresectable advanced sarcoma (locally advanced or metastatic) and having received at least one infusion of ICI (anti–PD‐1, anti–PD‐L1, anti‐CTLA4, or anti‐LAG3), were collected retrospectively. The diagnosis was pathologically confirmed by expert pathologists (RRePS Network). Treatment initiation had to occur before January 2023, within the framework of a clinical trial or compassionate use, across six different reference sarcoma centers in the NETSARC network (https://expertisesarcome.org/) in France from October 2015 to December 2023.

### Collected data

We retrospectively collected both clinical data (age, sex, Eastern Cooperative Oncology Group performance status [ECOG PS], history of autoimmune, immunosuppressive, or neoplastic diseases, corticosteroid use in the previous month, date of birth and death, date of last news, and vital status at last news), tumor characteristics (date of diagnosis, primary location, date of metastatic evolution, histological subtype, Fédération Nationale des Centres de Lutte Contre le Cancer [FNCLCC] grade, number and sites of metastasis at treatment initiation, previous therapeutic lines, PD‐L1 status, and TLS status according to Petitprez method^25^), pharmacological characteristics (type of ICI, concomitant treatment, date of first and last infusion, best objective response [OR], date of therapeutic switch, and reason for discontinuation), biological characteristics at treatment initiation (leukocyte count, total lymphocytes count, lactate dehydrogenase [LDH], C‐reactive protein [CRP], albuminemia, and derived neutrophil‐to‐lymphocyte ratio [dNLR] calculated as neutrophil/[leukocyte‐neutrophil] ratio), as well as information on the previous and subsequent line of therapy (date of initiation and progression and drug type).

The Lung Immune Prognostic Index (LIPI) was calculated using baseline dNLR >3 and LDH above the upper limit of normal, classifying patients into good (0 factor), intermediate (one factor), or poor (two factors) groups.

Sarcomas were categorized into simple genomic profile (few rearrangements, diploid karyotypes, and the presence of a specific oncogenic molecular alteration pathognomonic of the subtype such as fusion genes) or complex genomic profiles (highly rearranged sarcomas with multiple copy number alterations and mutations in tumor suppressor genes that contribute to the oncogenic mechanism).^26^

For a subset, NGS assessed tumor mutational burden (TMB) and key gene alterations. All variants that were not classified as variants of unknown significance and were present in more than one sample were selected for further analyses: *TP53*, *RB1*, *DNMT3A*, *PIK3CA*, *TERT*, *ATM*, *ATRX*, *CDKN2A/B*, and *PTEN*.

The panels used were Foundation Medicine, SureSelect CD Curie CGP panel (Agilent), and ProfilER.^27^ For a few patients, whole genome sequencing/whole exome sequencing and RNA sequencing (RNA‐seq) data were available within the Auragen national sequencing platform.

### Main objective

The aim of this study was to identify clinical and biological factors associated with ICI efficacy (PFS and OS).

### Statistical analyses

Quantitative variables were described using the number of observations, median, and first and third quartile values. Qualitative variables were described using the number of observations and percentage distribution. The number of missing data was presented but not considered for the percentage calculation.

OS was defined as the time from ICI initiation to death from any cause, and PFS was defined as the time from ICI initiation to the first disease progression or death. Patients who were alive were censored at their last known alive date.

Objective response rate (ORR) was defined as the percentage of patients who achieve a response, which can either be complete response (CR) or partial response (PR) according to Response Evaluation Criteria in Solid Tumors (RECIST).

OS and PFS curves were estimated using the Kaplan–Meier method and described using the median and 95% confidence interval (CI). The association between baseline characteristics and survival was estimated using the Cox proportional hazards regression model. Variables with fewer than 10% missing values and a *p* value <.15 in univariate models were considered. The following prespecified variables were forced into the model: age, primary tumor location, ICI type, and associated ICI. Subsequently, a backward selection using the Akaike Information Criterion was performed. The proportional hazard assumption was tested for all variables used in the Cox model using Schoenfeld residuals plots.

All tests were two‐sided and a *p* value <.05 was considered statistically significant. The analysis was performed using R software version 4.1.0.

### Regulatory aspects

In compliance with French ethical laws, patients were informed about the scientific use of their coded data; a nonopposition form was sent to those not previously notified. The study was conducted under the French National Commission for Informatics and Liberties reference methodology MR‐004 (declaration number: R201‐004‐302) and validated by a data protection officer in accordance with the General Data Protection Regulation (Regulation EU 2016/679). It was registered in the Centre Léon Bérard retrospective study registry (ET23000048, declaration R201‐004‐302) and approved by the Clinical Studies Review Committee in March 2023.

## RESULTS

### Patient characteristics

A total of 272 patients were included in the six participant centers, of which 127 (47%) were women, with a median age of 56 years (interquartile range [IQR], 44–65) (Table 1). Patients were in good general condition (ECOG PS 0‐1) in 95% of cases and the majority was heavily pretreated (38% had three or more prior lines of systemic therapy and 25% had two or more). Treatment was part of a clinical trial in 96% of cases.

**TABLE 1:** Baseline characteristics.

Characteristics of study population	
Age (years), median (min–max)	56 (15–88)
Sex, No. (%)	
Female	127 (47)
Male	145 (53)
ECOG PS, No. (%)	
0	104 (38)
1	154 (57)
≥2	14 (5)
Institution, No. (%)	
Centre Léon Bérard (Lyon)	69 (25)
Institut Gustave Roussy (Villejuif)	92 (34)
Institut Curie (Paris)	22 (8)
IUCT Oncopôle (Toulouse)	44 (16)
Centre Oscar Lambret (Lille)	32 (12)
Institut de Cancérologie de l’Ouest (Nantes)	13 (5)
Prior history, No. (%)	
Auto‐immune disease	2 (1)
Immune suppression	0 (0)
Neoplasia	25 (9)
Corticosteroid therapy in the month preceding initiation of treatment	16 (6)
Biological characteristics at treatment initiation, median (IQR)	
Leukocytes (G/L)	6.35 (5.06–8.37)
Neutrophils (G/L)	4.3 (3.03–6.07)
Lymphocytes (G/L)	1.2 (0.84–1.58)
<0.7 G/L, No. (%)	47 (17)
<1 G/L, No. (%)	94 (35)
dNLR	2.2 (1.52–2.96)
C‐reactive protein (mg/L)	15.8 (5–64)
Unknown, No. (%)	158 (58)
Albumin (g/L)	40 (34–44)
Unknown, No. (%)	15 (6)
Lactate deshydrogenase (IU/L)	229 (175–321)
Unknown, No. (%)	56 (21)
No. of previous lines of systemic therapy in advanced phase, No. (%)	
0	25 (9)
1	78 (29)
2	67 (25)
3 and more	102 (37)
Median	2 (min 0–max 10)
Tumor characteristics	
Stage at diagnosis, No. (%)	
Localized	152 (56)
Locally advanced	28 (10)
Metastatic	93 (34)
Stage at treatment initiation, No. (%)	
Locally advanced	16 (6)
Metastatic	257 (94)
Histotype, No. (%)	
Bone sarcoma	36 (13)
Chordoma	23 (8)
Osteosarcoma	7 (3)
Chondrosarcoma	5 (2)
Ewing sarcoma	1 (<1)
Parachordoma	1 (<1)
Soft tissue sarcoma	236 (87)
Leiomyosarcoma	46 (17)
Gastro‐intestinal stromal tumor	28 (10)
Liposarcoma	26 (10)
Dedifferentiated	17 (6)
Myxoid	7 (3)
Pleomorphic	2 (<1)
Undifferentiated pleomorphic sarcoma	20 (7)
Angiosarcoma	11 (4)
Malignant peripheral nerve sheath tumor	11 (4)
Fibrous solitary tumor	10 (4)
Alveolar soft part sarcoma	10 (4)
Epithelioid sarcoma	10 (4)
Rhabdomyosarcoma	9 (3)
Pleomorphic	6 (2)
Alveolar	2 (<1)
Epithelioid	1 (<1)
Desmoplastic small round cell tumor	7 (3)
SMARCA4‐deficient tumor	6 (2)
Synovial sarcoma	6 (2)
Clear cell sarcoma	4 (1)
Low grade fibromyxoid sarcoma	4 (1)
Epithelioid hemangioendothelioma	3 (1)
Rhabdoid tumor	2 (<1)
CIC‐rearranged sarcoma	2 (<1)
PEComa	2 (<1)
Endometrial stromal sarcoma	2 (<1)
Myxofibrosarcoma	2 (<1)
Intimal sarcoma	1 (<1)
Other	11 (4)
Genomic profile, No. (%)	
Simple	90 (33)
Complex	182 (67)
Primary tumor location, No. (%)	
Thorax	42 (15)
Abdomen and pelvis	141 (52)
Head and neck	16 (6)
Limb	72 (26)
Unknown	1 (<1)
FNLCLCC grade, No. (%)	
1	18 (7)
2	54 (20)
3	85 (31)
Unknown/not applicable	115 (42)
No. of metastatic sites, No. (%)	
0	16 (6)
1	76 (28)
2	93 (34)
≥3	87 (32)
Location of metastatic sites, No. (%)	
Liver	74 (27)
Lung	167 (61)
Peritoneum	80 (29)
Brain	11 (4)
Bone	70 (26)
Soft tissue (cutaneous, subcutaneous, fatty, muscle)	72 (26)
Lymph node	81 (30)
Adrenal gland	12 (4)
Pancreas	11 (4)
Molecular abnormalities, No. (%)	
*P53*	41/120 (34)
*Rb1*	18/115 (16)
*CDKN2A*	13/118 (11)
*ATRX*	11/106 (10)
*CDKN2B*	9/108 (8)
*PTEN*	10/117 (8)
*NF1*	8/109 (7)
*DNMT3A*	7/105 (7)
*ATM*	5/108 (5)
*PIK3CA*	5/114 (4)
*TERT* promoter	3/109 (3)
Presence of TLS	24/42 tested (57)
Tumor mutational burden (mutation/Mb), data available for No. (%)	98 (36)
Median (min–max)	2.52 (0–27)
Treatment‐related characteristics	
Type of ICI, No. (%)	
Anti–PD‐1	144 (53)
Anti–PD‐L1	128 (47)
Anti‐CTLA4	27 (10)
Anti‐LAG3	10 (4)
Associated treatment, No. (%)	
None	59 (22)
Cyclophosphamide	44 (16)
ICI	37 (14)
Olaparib	4 (1)
Regorafenib	36 (13)
Cobimetinib	47 (17)
Imatinib	2 (1)
Radiotherapy	38 (14)
Other	4 (1)
Treatment received in the context of a clinical trial, No. (%)	
Yes	261 (96)
No	11 (4)

Abbreviations: ECOG PS, Eastern Cooperative Oncology Group performance status; FNCLCC, Fédération Nationale des Centres de Lutte Contre le Cancer; ICI, immune checkpoint inhibitor; IQR, interquartile range; TLS, tertiary lymphoid structure.

Anti–PD‐1 and anti–PD‐L1 therapies were used in 53% (*n* = 144) and 47% (*n* = 128) of patients, respectively; 22% (*n* = 59) received monotherapy, whereas 31% (*n* = 85) had TKI combinations and 14% (*n* = 37) received dual ICI.

STS were predominant (*n* = 237, 87%). Leiomyosarcoma (LMS) was the most common histological subtype (*n* = 46, 17%), followed by gastro‐intestinal stromal tumor (GIST) (*n* = 28, 10%) and liposarcoma (*n* = 26, 10%). The other subtypes encountered are described in Table 1, with 10 (4%) ASPS and six (2%) SMARCA4‐deficient tumors.

The FNCLCC grade was known and applicable in 157 patients (58%), with a majority of grade 2 (20%) and grade 3 (31%). Most patients (94%) had distant metastases at treatment initiation, with 34% presenting with metastases at initial diagnosis. The most frequent sites were lungs (*n =* 167, 61%), lymph nodes (*n* = 81, 30%), peritoneum (*n* = 80, 29%), liver (*n* = 74, 27%), and bones and the soft tissues (*n* = 70 and *n* = 72, 26%).

At the time of treatment initiation, median CRP was 15.8 mg/L (IQR, 5–64), median dNLR 2.2 (IQR, 1.52–2.96), median albuminemia 40 g/L (IQR, 34–44), and median LDH 229 IU/L (IQR 175–321). Ninety‐four patients (35%) had a total lymphocyte count below 1 G/L.

Approximately two‐thirds of the patients had a complex genomic profile (*n* = 182, 67%).

Broad‐panel RNA‐seq data with estimated TMB was available for a third of the patients (33%, *n* = 91). The most frequently mutated gene was *p53* (37%, *n* = 34/91), followed by *Rb1* (19%, *n* = 17/91), *ATRX* (11%, *n* = 10/91), and *PTEN* (9.9%, *n* = 9/91).

### Treatment outcomes

At the time of data collection, the median follow‐up was 10.3 months (IQR, 4.6–19), and seven patients were not evaluable per RECIST 1.1. A total of 175 patients (64%) had died.

Efficacy data are summarized in Table S1. Among all patients evaluable, there were three CR (1.1%) and 43 PR (16%), resulting in an ORR of 17% (46/272). Eighty‐eight patients (33%) had stable disease (SD) as best response, whereas the other 125 patients (47%) had progressive disease (PD). Disease control rate (DCR) for the entire cohort was 49% at 3 months.

**FIGURE 1:**
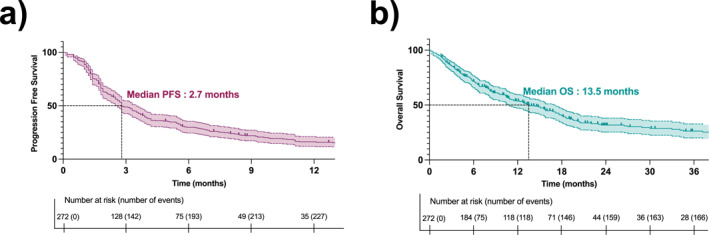
Kaplan–Meier curves of (A) progression‐free survival of sarcoma patients treated with ICI. (B) Overall survival of sarcoma patients treated with ICI (*n* = 272).

Median PFS was 2.7 months (95% CI, 2.5–3.4) and median OS was 13.5 months (95% CI, 11.0–17.3) (Figure 1). PFS curves by histotype for all histotypes with *n* > 10 patients are available in Figure S1.

Among responders (*n* = 46), the median duration of response was 14.5 months. The median time to response was 3.6 months. The following histotypes presented an OR: six of 10 ASPS (60%), five of 28 GIST (18%), four of 23 chordomas (17%), four of 10 epithelioid sarcomas (40%), four of 46 LMS (9%), four of 20 undifferentiated pleomorphic sarcoma (UPS) (20%), three of six SMARCA4‐deficient tumors (50%), two of 10 SFT (20%), two of 17 dedifferentiated liposarcoma (DDLPS) (12%), two of six pleomorphic rhabdomyosarcoma (RMS) (33%), one of 11 MPNST (9%), one of two myxofibrosarcoma (50%), one of seven osteosarcoma (14%), one of two PEComa (50%), one of one epithelioid RMS (100%), one of four low‐grade fibromyxoid sarcoma (25%), one of six synovial sarcoma (17%), one of seven desmoplastic small round cell tumor (14%), and one unclassified sarcoma (Figure S2).

### Variables associated with PFS

In multivariate analysis, ECOG PS 1 versus 0 (HR, 1.52; 95% CI, 1.14–2.04; *p* = .015), concomitant treatment with cyclophosphamide (HR, 2.09; 95% CI, 1.36–3.22; *p* = .013) or with MEK inhibitors (HR, 1.61; 95% CI, 1.05–2.47; *p* = .013), and high dNLR (HR, 1.18; 95% CI, 1.07–1.31; *p* = .001) were associated with a shorter PFS. ASPS was linked to better outcomes (median PFS, 25.85 months; HR, 0.38; 95% CI, 0.17–0.88; *p* = .036), whereas DDLPS was associated with poorer outcomes (median PFS, 2.4 months; HR, 1.92; 95% CI, 1.04–3.57; *p* = .036).

**FIGURE 2:**
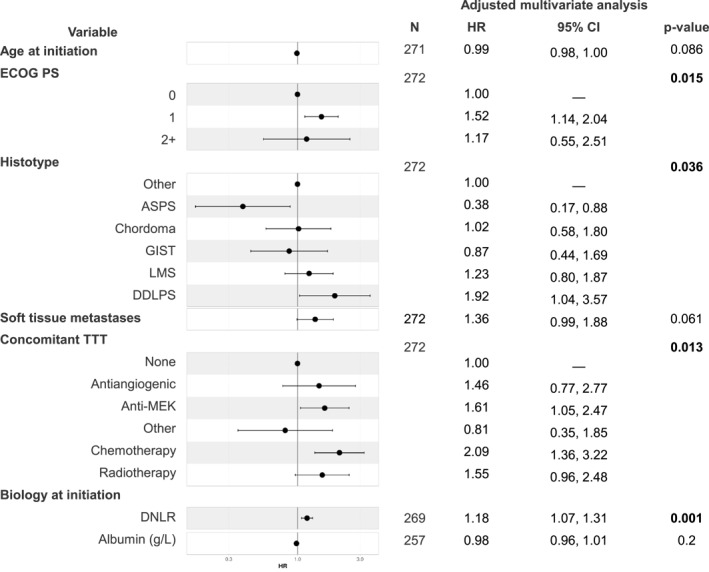
Cox regression multivariate analysis of variables associated with progression‐free survival. ASPS indicates alveolar soft part sarcoma; CI, confidence interval; concomitant TTT, concomitant treatment; DDLPS, dedifferentiated liposarcoma; dNLR, derived neutrophil lymphocyte ratio; ECOG PS, Eastern Cooperative Oncology Group Performance Status; GIST, gastro‐intestinal stromal tumor; HR, hazard ratio; LMS, leiomyosarcoma.

Notably, the presence of TLS was not included in the multivariate analysis due to the limited number of patients with available data (*n* = 42). The median PFS was not significantly different between the two groups (*p* = 0.1676) in univariate analysis, with 4.85 months in the 18 TLS– patients, compared to 2.6 months in the 24 TLS+ patients (Table S2; Figure 2). The ORR was 25% (six of 24) in TLS+ patients and 28% (five of 18) in TLS– patients.

### Variables associated with OS

In the multivariate analysis, DDLPS histotype (HR, 4.32; 95% CI, 2.07–9.03; *p* < .001), ECOG PS 1 versus 0 (HR, 2.22; 95% CI, 1.50–3.30; *p* < .001), concomitant cyclophosphamide (HR, 2.33; 95% CI, 1.33–4.10; *p* = .011), and elevated dNLR (HR, 1.39; 95% CI, 1.25–1.54; *p* < .001) were confirmed to be predictive of a poorer OS, whereas ASPS histotype (HR, 0.25; 95% CI, 0.07–0.86; *p* < .001) was associated with a better outcome. Notably, patients achieving a PR or CR had significantly longer OS (median OS, 50.87 months; 95% CI, 26.16–not reached) compared to those with SD (median OS, 20.10 months; 95% CI, 16.97–37.8) or PD (median OS 6.58 months; 95% CI, 5.81–9.8).

**FIGURE 3:**
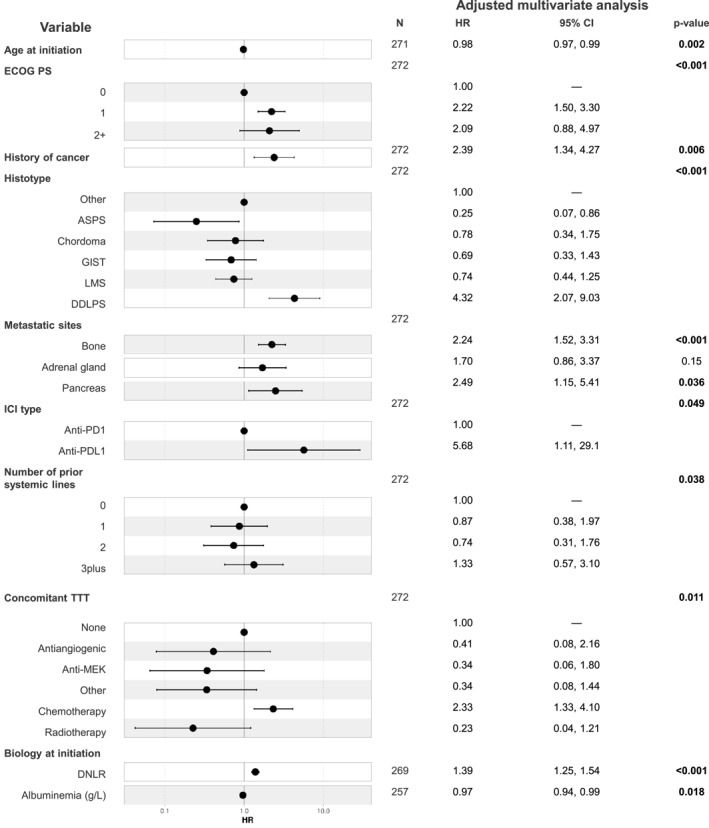
Cox regression multivariate analysis of variables associated with overall survival. ASPS, alveolar soft part sarcoma; concomitant TTT, concomitant treatment; CI, confidence interval; DDLS, dedifferentiated liposarcoma; dNLR, derived neutrophil lymphocyte ratio; ECOG PS, Eastern Cooperative Oncology Group Performance Status; GIST, gastro‐intestinal stromal tumor; HR, hazard ratio; LMS, leiomyosarcoma.

The use of anti–PD‐L1 was associated with a shorter OS compared to anti–PD‐1 therapy (HR, 5.68; 95% CI, 1.11–29.1; *p* = .049). Age at treatment initiation (HR, 0.98; 95% CI, 0.97–0.99; *p* = .002), history of neoplasia (HR, 2.39; 95% CI, 1.34–4.27; *p* = .006), bone metastases (HR, 2.24; 95% CI, 1.52–3.31; *p* < .001), and pancreatic metastases (HR, 2.49; 95% CI, 1.15–5.41; *p* = .036) were associated with OS (Table S3; Figure 3).

### Long responders

**FIGURE 4:**
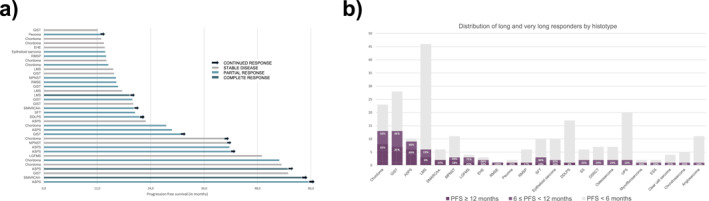
Description of long‐responder sarcoma patients to immune checkpoint inhibitors at time of data cutoff. (A) Swimmer plot illustrating the PFS in months of very‐long responders (PFS >12 months). (B) Distribution of long (PFS >6 months) and very long responders (PFS >12 months) among each histotype. Each bar in the chart represents the total number of patients for each histotype. The light purple segment indicates the proportion of long responders (PFS >6 months). Within this segment, the striped portion represents the subset of very long responders (PFS >12 months). ASPS, alveolar soft part sarcoma; DDLS, dedifferentiated liposarcoma; DSRCT, desmoplastic small round cell tumor; EHE, epithelioid hemangioendothelioma; ESS, endometrial stromal sarcoma; GIST, gastro‐intestinal stromal tumor; LGFMS, low‐grade fibromyxoid sarcoma; LMS, leiomyosarcoma; MPNST, malignant peripheral nerve sheath tumor; PFS, progression‐free survival; RMSE, embryonal rhabdomyosarcoma; RMSP, pleomorphic rhabdomyosarcoma; SFT, solitary fibrous tumor; SS, synovial sarcoma; UPS, undifferentiated pleomorphic sarcoma.

Seventy‐five patients (28%) had a PFS >6 months (of which 38 [51%] with CR or PR as best response), whereas 36 patients (13%) had a PFS >12 months (of which 21 [58%] with CR or PR as best response) (Figure 4A). Twenty‐three histological subtypes presented with either a long (PFS >6 months) or a very long (PFS >12 months) response (Figure 4B). The following factors were associated with PFS >6 months: ASPS (OR, 3.99; 95% CI, 1.46–10.8; *p* < .001) and chordoma subtypes (OR, 1.11; 95% CI, 1.35–6.60; *p* < .001) and low dNLR (OR, 0.78; 95% CI, 0.61–.0.97; *p* = .022) (Table S4).

### Subset analysis: Molecular alterations

In a multivariate analysis investigating PFS on the 91 patients with complete molecular data, *NF1* mutation (HR, 0.13; 95% CI, 0.03–0.53; *p* < .001) was associated with a favorable outcome whereas *RB1* mutation (HR, 2.08; 95% CI, 1.12–3.84; *p* = .025) was linked to a poorer PFS (Table S5; Figure S3).

### Subset analysis: LIPI score

Baseline dNLR >3 and LDH > ULN, both independently linked to OS, were combined to calculate the LIPI. Among 216 patients, 94 (44%) had good LIPI scores, 92 (43%) had intermediate LIPI scores, and 30 (14%) had poor LIPI scores.

Median OS was 19.1 months (good), 11.8 months (intermediate; HR, 1.56; 95% CI, 1.03–2.36), and 2.15 months (poor; HR, 3.57; 95% CI, 2.05–6.21; *p* < .001). Median PFS was 3.9, 2.8 (HR, 1.24; 95% CI, 0.90–1.70), and 1.1 months (HR, 2.24; 95% CI, 1.39–3.60; *p* = .006), respectively (Figure S4).

### Safety

Overall, 151 patients (56%) developed any grade toxicity due to ICI (Table S6). The most common toxicities were dermatological (24%; mostly rash and pruritus), and endocrine immune‐related adverse events (irAEs) (21%; mostly hypothyroidism, one case of hypophysitis and three cases of diabetes). Sixteen patients (5%) developed irAEs of CTCAE grade 3 or higher; including three patients with grade 3 colitis, three patients with grade 3 hepatitis, three patients with grade 3 myocarditis, one patient with grade 3 infusion related reaction, one patient with grade 3 rash, two patients with grade 4 diabetic ketoacidosis, and three patients who had asymptomatic grade 4 creatine phosphokinase elevation. There was no grade 5.

In total, 26 patients (10%) discontinued ICI therapy due to toxicity.

## DISCUSSION

To our knowledge, this study represents one of the largest retrospective analyses evaluating the use of ICIs in advanced sarcomas. Drawing from a multi‐institutional cohort across six reference centers, our study offers insights into the efficacy and safety of ICIs across a broad range of histological subtypes.

Overall, ICI therapy was well tolerated, consistent with previous studies. We observed an ORR of 17% and a DCR of 49%. Importantly, responses were often durable, with 28% of patients achieving a PFS exceeding 6 months and 13% exceeding 12 months. These findings highlight the potential of ICI to address a critical unmet need in this patient population, particularly given the limited efficacy of conventional cytotoxic chemotherapy.

Among the 25 treatment‐naive patients (9%), eight (32%) achieved a PFS >12 months, with most prolonged responses seen in GIST, DDLPS, LMS, and UPS—histotypes not typically considered immune‐sensitive. Notably, only one ASPS was included in this subgroup, indicating that the results were not driven by this classically ICI‐sensitive tumor. OR occurred in seven patients (28%), and 11 patients (44%) achieved SD. Regarding treatment regimens, 14 patients received ICI monotherapy, eight received dual ICI combinations, and three received ICI plus radiotherapy. As observed in other tumor types,^28^, ^29^, ^30^ these results support the potential benefit of frontline ICI before prior treatments may alter the tumor microenvironment. However, the study was underpowered to detect a significant survival difference.

Histological subtype emerged as a key determinant of ICI efficacy. Consistent with previous reports, ASPS and chordomas demonstrated high sensitivity to immunotherapy. Conversely, DDLPS were frequently refractory, likely due to mechanisms such as MDM2 amplification, which has been associated with reduced immunogenicity and hyperprogression.^31^ Interestingly, 46% of GIST patients in our cohort experienced PFS >6 months (13 of 75 long responders), and 25% exceeded 12 months (seven of 36 very long responders). Three of them were *KIT*/*PDGFRA* wild‐type GIST, and the others had *KIT* exon 11 mutations. All GIST responders received ICI in combination with other agents. Eleven also received regorafenib, which should be contextualized with the median PFS of 4.8 months as a third‐line treatment observed in the GRID trial.^32^ The longest response was observed in a patient treated with pembrolizumab and cyclophosphamide, suggesting that the clinical benefit was largely driven by immunotherapy, given the limited efficacy of chemotherapy in this context. Additionally, one patient received imatinib as a rechallenge after progressing on imatinib, sunitinib, and regorafenib, with a median PFS of 1.8 months typically expected in such cases.^33^

Prolonged responses were observed in chordomas and SMARCA4‐deficient tumors. Among chordomas, 57% had a PFS exceeding 6 months and 35% exceeded 12 months. Two of six SMARCA4‐deficient tumors were very long responders, further supporting the potential role of immunotherapy in select molecular subtypes.

ECOG PS was also associated with outcomes. However, given that 96% of patients had PS 0–1 due to clinical trial inclusion criteria, our ability to assess differences in patients with PS ≥2 was limited.

We observed inferior OS with anti–PD‐L1 therapy compared to anti–PD‐1 therapy, a trend also reported in other malignancies.^34^ This may be explained by the broader inhibition profile of anti–PD‐1 agents, which block both PD‐L1 and PD‐L2 interactions.^35^ However, this OS difference was not mirrored in PFS, potentially due to the high degree of heterogeneity or the delayed clinical benefit of ICIs.

Combination regimens also had a significant impact on outcomes. Patients receiving pembrolizumab plus cyclophosphamide (PEMBROSARC cohort) experienced worse outcomes, potentially due to T‐cell lymphopenia, a known effect of cyclophosphamide. Similarly, the combination of pembrolizumab and cobimetinib was associated with shorter PFS, aligning with earlier studies that highlight the critical role of MAPK signaling in naive T‐cell priming^36^ and interleukin production.^37^ These findings raise important concerns about the selection of agents used in immunotherapy combinations. Although combination strategies are designed to enhance efficacy by modifying the tumor microenvironment, the lack of benefit in our study may reflect suboptimal drug pairings or increased toxicity. In contrast, recent single‐arm trials combining ICIs with cytotoxic chemotherapy have reported encouraging results, supporting the continued evaluation of rationally designed combinations.^38^

Systemic inflammation, as measured by the LIPI, was associated with worse outcomes. Although data were incomplete, higher LIPI scores correlated with poorer survival. However, without a control arm of patients not treated with ICIs, it is difficult to determine whether LIPI is predictive of ICI benefit or merely prognostic.

The presence of TLS did not correlate with improved outcomes in our cohort. The data was available for only 42 patients (15%). This finding contrasts with previous reports in other cancers and may reflect histotype‐specific differences in the immune microenvironment.

TMB was found to be correlated to response to immunotherapy in some specific subtypes of sarcomas.^39^ It was generally low in our cohort, consistent with The Cancer Genome Atlas data, although responses were seen in certain subtypes with higher TMB.

Subgroup analyses revealed a potential association between *NF1* mutations and improved ICI efficacy, in line with findings in lung adenocarcinoma.^40^ However, *NF1* mutations were identified in only eight patients—five malignant peripheral nerve sheath tumor (MPNST), two RMS (epithelioid and pleomorphic), and one UPS. Conversely, *RB1* mutations were linked to poorer outcomes, as reported in non–small cell lung and bladder cancers.^41^, ^42^ However, these associations were based on a limited number of cases and should be interpreted with caution pending further molecular profiling across the full cohort.

This study has several limitations. Its retrospective nature and histological heterogeneity limited statistical power for subgroup analyses. Histological grade could not be included in multivariate models due to missing data, especially in small, metastatic, or pretreated samples, or in histotypes where FNCLCC grading is not applicable (e.g., GIST, bone sarcomas). Molecular data, including TMB and key mutations, were available for only 91 patients and were primarily derived from RNA‐seq; these findings require validation with DNA‐based methods in the whole cohort.

The study population was enriched for histological subtypes prioritized for inclusion in immunotherapy trials, potentially introducing selection bias. This may explain the high incidence of radiologically identified lymph node metastases, which are rare in sarcomas. Finally, distinguishing ICI‐related disease control from the naturally indolent course of certain subtypes (e.g., chordoma, wild‐type GIST) was challenging. Although we considered using prior‐line PFS as a comparator, this information was unavailable for a substantial proportion of patients (*n* = 45).

In conclusion, this retrospective, multi‐institutional study identified clinical and biological factors associated with the efficacy of ICIs in sarcomas, including novel histological subtypes and specific molecular alterations, such as *NF1*. Overall, these findings support the efficacy of ICIs in treating some specific histological and molecular subsets of advanced sarcomas.

## AUTHOR CONTRIBUTIONS

**Mina Fazel**: Conceptualization, investigation, writing– original draft, writing– review and editing, methodology, validation, and project administration. **Romain Varnier**: Methodology. **Hélène Vanacker**: Writing– review and editing. **Nicolas Penel**: Writing– review and editing and investigation. **Sarah Watson**: Writing– review and editing. **Benjamin Verret**: Writing– review and editing. **Thibaud Valentin**: Writing– review and editing. **Emmanuelle Bompas**: Writing– review and editing. **Waisse Waissi**: Writing– review and editing. **Alexandra Meurgey**: Writing– review and editing. **Françoise Ducimetière**: Writing– review and editing. **Jean‐Yves Blay**: Supervision, writing– review and editing, conceptualization, methodology, and validation. **Armelle Dufresne**: Writing– review and editing, supervision, conceptualization, methodology, and validation. **Mehdi Brahmi**: Conceptualization, methodology, validation, writing– review and editing, supervision, and project administration.

## CONFLICT OF INTEREST STATEMENT

Jean‐Yves Blay reports fees for professional activities from Institut National Du Cancer; and grant and/or contract funding from Institut National Du Cancer. Emmanuelle Bompas reports fees for professional activities from ico. Mehdi Brahmi reports fees for professional activities from Deciphera Pharmaceuticals; and travel fees from PharmaMar. Benjamin Verret reports consulting fees from Lilly (to my institution), Pfizer (to my institution), Netcancer, Pierre Fabre, Seagen, Daichii Sankyo (to my institution), Gilead (to my institution), Novartis (to my institution), MSD (to my institution) AZ (to my institution), Owkins (to my institution), Boehringer Ingelheim (to my institution); and travel expenses from Lilly, Novartis, Pfizer, Accord Healthcare, Amgen, AZ, Daichii Sankyo, F. Hoffmann‐La Roche AG, and AstraZeneca. Sarah Watson reports fees for professional activities from Boehringer Ingelheim, Deciphera Pharmaceuticals, and PharmaMar. Armelle Dufresne reports consulting fees from Springworks; fees for professional activities from Deciphera; travel funds from Pharmamar and Pfizer; and grant and/or contract funding from Bayer, GSK, and Adaptimmune. The other authors declare no conflicts of interest.

## Supporting information

Supplementary Material

## Data Availability

De‐identified participant data are available on reasonable request (mina.fazel@lyon.unicancer.fr).
