# Supplementary material for: Clinical and biological factors associated with response to immune checkpoint inhibitors in advanced sarcomas: IMPRESARC, a French retrospective multicenter cohort study
Source: Cancer. 2025 Sep 5;131(18):e70052. doi: 10.1002/cncr.70052 (PMC12412778; doi:10.1002/cncr.70052)
Supplement: Supplementary file 1 — Supplementary Material [file CNCR-131-e70052-s001.docx]

**Supplemental Material**

| Best overall response – n (%) – 265 evaluable patients | |
| --- | --- |
| Complete response (CR) | 3 (1.1%) |
| Partial response (PR) | 43 (16%) |
| Stable disease (SD) | 88 (33%) |
| Progressive disease (PD) | 125 (47%) |
| Dissociated response (DR) | 6 (2.3%) |
| Not evaluable | 7 (2.6%) |
| Objective response rate (ORR = PR+CR) | 46 (17%) |
| Disease control rate (DCR = PR+CR+SD) | 134 (49%) |

**Supplementary Table 1.** **Efficacy**

**Supplementary Figure 1. Kaplan-Meier curves of progression-free survival of sarcoma patients treated with ICI by histotype (n>10).** ASPS = alveolar soft part sarcoma, DDLPS = dedifferentiated liposarcoma, GIST = gastro-intestinal stromal tumor, LMS = leiomyosarcoma, mPFS = median progression-free survival, MPNST = malignant peripheral nerve sheath tumor, PFS = progression-free survival, SFT = solitary fibrous tumor, UPS = undifferentiated pleomorphic sarcoma.

**Supplementary Figure 2. Efficacy data by histotype in histological subtypes with at least 1 objective response.** The percentages represent the objective response rate by histological subtype. ASPS = alveolar soft part sarcoma, DDLPS = dedifferentiated liposarcoma, DSRCT = desmoplastic small round cell tumor, ERMS = epithelioid rhabdomyosarcoma, GIST = gastro-intestinal stromal tumor, LGFMS = low-grade fibromyxoid sarcoma, LMS = leiomyosarcoma, MPNST = malignant peripheral nerve sheath tumor, PRMS = pleomorphic rhabdomyosarcoma, SFT = solitary fibrous tumor, SMARCA4- = SMARCA4-deficient tumor, UPS = undifferentiated pleomorphic sarcoma

| **Characteristic** | **Univariate analysis** | | | | **Adjusted multivariate analysis** | | |
| --- | --- | --- | --- | --- | --- | --- | --- |
|  | **N** | **HR** | **95% CI** | **p-value** | **HR** | **95% CI** | **p-value** |
| **Age at initiation** | 271 | 1.00 | 0.99, 1.01 | 0.7 | 0.99 | 0.98, 1.00 | 0.086 |
| **Sex** | 272 |  |  | >0.9 |  |  |  |
| Female |  | 1.00 | — |  |  |  |  |
| Male |  | 0.99 | 0.77, 1.27 |  |  |  |  |
| **ECOG PS** | 272 |  |  | <0.001 |  |  | 0.015 |
| 0 |  | 1.00 | — |  | 1.00 | — |  |
| 1 |  | 1.66 | 1.27, 2.16 |  | 1.52 | 1.14, 2.04 |  |
| 2+ |  | 1.93 | 1.07, 3.47 |  | 1.17 | 0.55, 2.51 |  |
| **AI disease** | 272 | 1.47 | 0.55, 3.97 | 0.5 |  |  |  |
| **History of cancer** | 272 | 1.34 | 0.87, 2.05 | 0.2 |  |  |  |
| **Corticosteroid** | 272 | 0.68 | 0.40, 1.17 | 0.14 |  |  |  |
| **Location** | 271 |  |  | >0.9 |  |  |  |
| Abdomen/pelvis |  | 1.00 | — |  |  |  |  |
| Limb |  | 1.01 | 0.75, 1.36 |  |  |  |  |
| Head/neck |  | 0.99 | 0.59, 1.67 |  |  |  |  |
| Thorax |  | 1.08 | 0.75, 1.57 |  |  |  |  |
| **Histotype** | 272 |  |  | <0.001 |  |  | 0.036 |
| Other |  | 1.00 | — |  | 1.00 | — |  |
| ASPS |  | 0.22 | 0.10, 0.48 |  | 0.38 | 0.17, 0.88 |  |
| Chordoma |  | 0.50 | 0.32, 0.79 |  | 1.02 | 0.58, 1.80 |  |
| GIST |  | 0.64 | 0.42, 0.98 |  | 0.87 | 0.44, 1.69 |  |
| LMS |  | 1.12 | 0.79, 1.57 |  | 1.23 | 0.80, 1.87 |  |
| DDLPS |  | 1.14 | 0.68, 1.92 |  | 1.92 | 1.04, 3.57 |  |
| **Genomic profile** | 272 |  |  | 0.010 |  |  |  |
| Complex |  | 1.00 | — |  |  |  |  |
| Simple |  | 0.71 | 0.54, 0.92 |  |  |  |  |
| **FNCLCC grade** | 157 |  |  | 0.5 |  |  |  |
| 1 |  | 1.00 | — |  |  |  |  |
| 2 |  | 0.82 | 0.47, 1.42 |  |  |  |  |
| 3 |  | 0.99 | 0.59, 1.68 |  |  |  |  |
| **Stage at diagnosis** | 272 |  |  | 0.4 |  |  |  |
| Localized |  | 1.00 | — |  |  |  |  |
| Locally advanced |  | 1.11 | 0.73, 1.69 |  |  |  |  |
| Metastatic |  | 0.85 | 0.64, 1.12 |  |  |  |  |
| **Stage at initiation** | 272 |  |  | 0.6 |  |  |  |
| Locoregional |  | 1.00 | — |  |  |  |  |
| Locally advanced |  | 0.87 | 0.25, 3.10 |  |  |  |  |
| Metastatic |  | 1.13 | 0.36, 3.55 |  |  |  |  |
| **NF1 mutation** | 109 | 0.52 | 0.24, 1.13 | 0.071 |  |  |  |
| **PDL1 expression** | 99 | 3.74 | 0.50, 27.7 | 0.3 |  |  |  |
| **P53 mutation** | 120 | 1.19 | 0.80, 1.76 | 0.4 |  |  |  |
| **RB1 mutation** | 115 | 2.39 | 1.41, 4.06 | 0.003 |  |  |  |
| **DNMT3 mutation** | 105 | 0.57 | 0.25, 1.31 | 0.2 |  |  |  |
| **PIK3CA mutation** | 114 | 0.75 | 0.30, 1.85 | 0.5 |  |  |  |
| **TERT mutation** | 109 | 0.80 | 0.25, 2.53 | 0.7 |  |  |  |
| **AMT mutation** | 108 | 0.89 | 0.33, 2.43 | 0.8 |  |  |  |
| **ATRX mutation** | 106 | 1.02 | 0.53, 1.98 | >0.9 |  |  |  |
| **CDKN2A mut** | 118 | 1.05 | 0.59, 1.88 | 0.9 |  |  |  |
| **CDKN2B mut** | 108 | 1.09 | 0.55, 2.17 | 0.8 |  |  |  |
| **PTEN mutation** | 117 | 0.98 | 0.49, 1.94 | >0.9 |  |  |  |
| **Presence of TLS** | 42 | 1.57 | 0.80, 3.08 | 0.2 |  |  |  |
| **TMB (mut/Mb)** | 98 |  |  | 0.3 |  |  |  |
| < 10 |  | 1.00 | — |  |  |  |  |
| ≥ 10 |  | 0.59 | 0.19, 1.89 |  |  |  |  |
| **Metastatic sites** | 272 |  |  | 0.14 |  |  |  |
| 0 |  | 1.00 | — |  |  |  |  |
| 1 |  | 1.03 | 0.59, 1.79 |  |  |  |  |
| 2 |  | 1.21 | 0.70, 2.10 |  |  |  |  |
| 3 and more |  | 1.48 | 0.85, 2.57 |  |  |  |  |
| **Lung** | 272 | 1.25 | 0.97, 1.62 | 0.084 |  |  |  |
| **Liver** | 272 | 1.06 | 0.80, 1.40 | 0.7 |  |  |  |
| **Peritoneum** | 272 | 1.09 | 0.83, 1.43 | 0.5 |  |  |  |
| **Brain** | 272 | 0.73 | 0.38, 1.37 | 0.3 |  |  |  |
| **Bone** | 272 | 1.43 | 1.08, 1.90 | 0.017 |  |  |  |
| **Soft tissue** | 272 | 1.38 | 1.04, 1.83 | 0.029 | 1.36 | 0.99, 1.88 | 0.061 |
| **Adrenal gland** | 272 | 1.38 | 0.77, 2.46 | 0.3 |  |  |  |
| **Pancreas** | 272 | 1.66 | 0.88, 3.13 | 0.15 |  |  |  |
| **Lymph node** | 272 | 1.44 | 1.10, 1.88 | 0.010 |  |  |  |
| **Other** | 272 | 1.00 | 0.62, 1.62 | >0.9 |  |  |  |
| **Number of prior**  **systemic lines** | 272 |  |  | 0.002 |  |  |  |
|  |  |  |  |  |  |  |  |
| 0 |  | 1.00 | — |  |  |  |  |
| 1 |  | 1.57 | 0.95, 2.58 |  |  |  |  |
| 2 |  | 1.83 | 1.10, 3.03 |  |  |  |  |
| 3plus |  | 2.27 | 1.40, 3.68 |  |  |  |  |
| **ICI type** | 272 |  |  | 0.047 |  |  |  |
| Anti-PD1 |  | 1.00 | — |  |  |  |  |
| Anti-PDL1 |  | 1.29 | 1.00, 1.66 |  |  |  |  |
| **Associated ICI** | 272 |  |  | 0.6 |  |  |  |
| None |  | 1.00 | — |  |  |  |  |
| Anti-CTLA4 |  | 0.81 | 0.53, 1.25 |  |  |  |  |
| Anti-LAG3 |  | 0.93 | 0.46, 1.89 |  |  |  |  |
| **Concomitant TTT** | 272 |  |  | <0.001 |  |  | 0.013 |
| None |  | 1.00 | — |  | 1.00 | — |  |
| Antiangiogenic |  | 1.20 | 0.81, 1.79 |  | 1.46 | 0.77, 2.77 |  |
| Anti-MEK |  | 1.99 | 1.37, 2.87 |  | 1.61 | 1.05, 2.47 |  |
| Other |  | 1.05 | 0.51, 2.18 |  | 0.81 | 0.35, 1.85 |  |
| Chemotherapy |  | 2.30 | 1.59, 3.33 |  | 2.09 | 1.36, 3.22 |  |
| Radiotherapy |  | 1.73 | 1.17, 2.55 |  | 1.55 | 0.96, 2.48 |  |
| **DNLR** | 269 | 1.16 | 1.07, 1.26 | <0.001 | 1.18 | 1.07, 1.31 | 0.001 |
| **LDH (UI/L)** | 216 | 1.00 | 1.00, 1.00 | 0.018 |  |  |  |
| **CRP (mg/L)** | 114 | 1.01 | 1.00, 1.01 | <0.001 |  |  |  |
| **Albumin (g/L)** | 257 | 0.96 | 0.94, 0.98 | <0.001 | 0.98 | 0.96, 1.01 | 0.2 |

**Supplementary Table 2. Univariate and multivariate analysis of variables associated with progression-free survival.** AI = autoimmune, ASPS = alveolar soft part sarcoma, concomitant TTT = concomitant treatment, 95% CI = 95% confidence interval, CRP = C-reactive protein, DDLS = dedifferentiated liposarcoma, DNLR = derived neutrophil lymphocyte ratio, ECOG PS = Eastern Cooperative Oncology Group Performance Status, FNCLCC = fédération nationale des centres de lutte contre le cancer, GIST = gastro-intestinal stromal tumor, HR = hazard ratio, ICI = immune checkpoint inhibitor, LDH = lactate dehydrogenase, LMS = leiomyosarcoma, TLS = tertiary lymphoid structure, TMB = tumor mutational burden.

| **Characteristic** | **Univariate analysis** | | | | **Adjusted multivariate analysis** | | |
| --- | --- | --- | --- | --- | --- | --- | --- |
|  | **N** | **HR** | **95% CI** | **p-value** | **HR** | **95% CI** | **p-value** |
| **Age at initiation** | 271 | 1.00 | 0.99, 1.01 | >0.9 | 0.98 | 0.97, 0.99 | 0.002 |
| **Sex** | 272 |  |  | 0.6 |  |  |  |
| Female |  | 1.00 | — |  |  |  |  |
| Male |  | 1.08 | 0.80, 1.46 |  |  |  |  |
| **ECOG PS** | 272 |  |  | <0.001 |  |  | <0.001 |
| 0 |  | 1.00 | — |  | 1.00 | — |  |
| 1 |  | 2.80 | 1.98, 3.94 |  | 2.22 | 1.50, 3.30 |  |
| 2+ |  | 5.22 | 2.73, 9.99 |  | 2.09 | 0.88, 4.97 |  |
| **AI disease** | 272 | 0.63 | 0.16, 2.56 | 0.5 |  |  |  |
| **History of cancer** | 272 | 1.86 | 1.15, 3.01 | 0.019 | 2.39 | 1.34, 4.27 | 0.006 |
| **Corticosteroid** | 272 | 0.88 | 0.46, 1.66 | 0.7 |  |  |  |
| **Location** | 271 |  |  | 0.13 |  |  |  |
| Abdomen/pelvis |  | 1.00 | — |  |  |  |  |
| Limb |  | 1.19 | 0.83, 1.70 |  |  |  |  |
| Head/neck |  | 1.41 | 0.76, 2.60 |  |  |  |  |
| Thorax |  | 1.67 | 1.09, 2.55 |  |  |  |  |
| **Histotype** | 272 |  |  | <0.001 |  |  | <0.001 |
| Other |  | 1.00 | — |  | 1.00 | — |  |
| ASPS |  | 0.15 | 0.05, 0.46 |  | 0.25 | 0.07, 0.86 |  |
| Chordoma |  | 0.28 | 0.14, 0.55 |  | 0.78 | 0.34, 1.75 |  |
| GIST |  | 0.59 | 0.35, 1.00 |  | 0.69 | 0.33, 1.43 |  |
| LMS |  | 0.69 | 0.45, 1.05 |  | 0.74 | 0.44, 1.25 |  |
| DDLPS |  | 1.24 | 0.68, 2.25 |  | 4.32 | 2.07, 9.03 |  |
| **Genomic profile** | 272 |  |  | 0.044 |  |  |  |
| Complex |  | 1.00 | — |  |  |  |  |
| Simple |  | 0.72 | 0.52, 1.00 |  |  |  |  |
| **FNCLCC grade** | 157 |  |  | 0.2 |  |  |  |
| 1 |  | 1.00 | — |  |  |  |  |
| 2 |  | 1.79 | 0.80, 4.00 |  |  |  |  |
| 3 |  | 1.96 | 0.90, 4.27 |  |  |  |  |
| **Stage at diagnosis** | 272 |  |  | 0.6 |  |  |  |
| Localized |  | 1.00 | — |  |  |  |  |
| Locally advanced |  | 1.29 | 0.78, 2.10 |  |  |  |  |
| Metastatic |  | 1.06 | 0.77, 1.47 |  |  |  |  |
| **Stage at initiation** | 272 |  |  | 0.003 |  |  |  |
| Locoregional |  | 1.00 | — |  |  |  |  |
| Locally advanced |  | 0.33 | 0.06, 2.00 |  |  |  |  |
| Metastatic |  | 1.52 | 0.38, 6.12 |  |  |  |  |
| **NF1 mutation** | 109 | 1.35 | 0.61, 2.96 | 0.5 |  |  |  |
| **PDL1 expression** | 99 | 7.62 | 0.99, 58.6 | 0.13 |  |  |  |
| **P53 mutation** | 120 | 1.10 | 0.69, 1.75 | 0.7 |  |  |  |
| **RB1 mutation** | 115 | 1.11 | 0.59, 2.12 | 0.7 |  |  |  |
| **DNMT3 mutation** | 105 | 0.19 | 0.03, 1.40 | 0.030 |  |  |  |
| **PIK3CA mutation** | 114 | 0.97 | 0.35, 2.68 | >0.9 |  |  |  |
| **TERT mutation** | 109 | 1.57 | 0.49, 5.02 | 0.5 |  |  |  |
| **AMT mutation** | 108 | 2.02 | 0.73, 5.61 | 0.2 |  |  |  |
| **ATRX mutation** | 106 | 0.89 | 0.40, 1.94 | 0.8 |  |  |  |
| **CDKN2A mut** | 118 | 1.04 | 0.52, 2.11 | >0.9 |  |  |  |
| **CDKN2B mut** | 108 | 1.07 | 0.46, 2.49 | 0.9 |  |  |  |
| **PTEN mutation** | 117 | 0.67 | 0.27, 1.66 | 0.4 |  |  |  |
| **Presence of TLS** | 42 | 1.66 | 0.76, 3.63 | 0.2 |  |  |  |
| **TMB (mut/Mb)** | 98 |  |  | >0.9 |  |  |  |
| < 10 |  | 1.00 | — |  |  |  |  |
| ≥ 10 |  | 0.93 | 0.23, 3.81 |  |  |  |  |
| **Metastatic sites** | 272 |  |  | <0.001 |  |  |  |
| 0 |  | 1.00 | — |  |  |  |  |
| 1 |  | 4.21 | 1.31, 13.6 |  |  |  |  |
| 2 |  | 4.89 | 1.53, 15.6 |  |  |  |  |
| 3 and more |  | 6.85 | 2.15, 21.9 |  |  |  |  |
| **Lung** | 272 | 1.60 | 1.16, 2.19 | 0.003 |  |  |  |
| **Liver** | 272 | 1.01 | 0.73, 1.40 | >0.9 |  |  |  |
| **Peritoneum** | 272 | 1.09 | 0.79, 1.51 | 0.6 |  |  |  |
| **Brain** | 272 | 0.94 | 0.46, 1.92 | 0.9 |  |  |  |
| **Bone** | 272 | 2.19 | 1.60, 3.02 | <0.001 | 2.24 | 1.52, 3.31 | <0.001 |
| **Soft tissue** | 272 | 1.21 | 0.87, 1.69 | 0.3 |  |  |  |
| **Adrenal gland** | 272 | 1.80 | 0.97, 3.31 | 0.084 | 1.70 | 0.86, 3.37 | 0.15 |
| **Pancreas** | 272 | 1.92 | 0.94, 3.92 | 0.10 | 2.49 | 1.15, 5.41 | 0.036 |
| **Lymph node** | 272 | 1.55 | 1.13, 2.12 | 0.007 |  |  |  |
| **Other** | 272 | 1.24 | 0.73, 2.10 | 0.4 |  |  |  |
| **Number of prior**  **systemic lines** | 272 |  |  | <0.001 |  |  | 0.038 |
|  |  |  |  |  |  |  |  |
| 0 |  | 1.00 | — |  | 1.00 | — |  |
| 1 |  | 1.84 | 0.90, 3.79 |  | 0.87 | 0.38, 1.97 |  |
| 2 |  | 1.86 | 0.91, 3.83 |  | 0.74 | 0.31, 1.76 |  |
| 3plus |  | 3.05 | 1.53, 6.09 |  | 1.33 | 0.57, 3.10 |  |
| **ICI type** | 272 |  |  | 0.12 |  |  | 0.049 |
| Anti-PD1 |  | 1.00 | — |  | 1.00 | — |  |
| Anti-PDL1 |  | 1.27 | 0.94, 1.73 |  | 5.68 | 1.11, 29.1 |  |
| **Associated ICI** | 272 |  |  | 0.4 |  |  |  |
| None |  | 1.00 | — |  |  |  |  |
| Anti-CTLA4 |  | 0.65 | 0.34, 1.23 |  |  |  |  |
| Anti-LAG3 |  | 1.10 | 0.45, 2.69 |  |  |  |  |
| **Concomitant TTT** | 272 |  |  | 0.001 |  |  | 0.011 |
| None |  | 1.00 | — |  | 1.00 | — |  |
| Antiangiogenic |  | 1.14 | 0.68, 1.89 |  | 0.41 | 0.08, 2.16 |  |
| Anti-MEK |  | 2.28 | 1.48, 3.51 |  | 0.34 | 0.06, 1.80 |  |
| Other |  | 0.76 | 0.27, 2.10 |  | 0.34 | 0.08, 1.44 |  |
| Chemotherapy |  | 1.94 | 1.27, 2.98 |  | 2.33 | 1.33, 4.10 |  |
| Radiotherapy |  | 1.35 | 0.82, 2.23 |  | 0.23 | 0.04, 1.21 |  |
| **DNLR** | 269 | 1.40 | 1.28, 1.53 | <0.001 | 1.39 | 1.25, 1.54 | <0.001 |
| **LDH (UI/L)** | 216 | 1.00 | 1.00, 1.00 | <0.001 |  |  |  |
| **CRP (mg/L)** | 114 | 1.01 | 1.01, 1.01 | <0.001 |  |  |  |
| **Albumin (g/L)** | 257 | 0.93 | 0.91, 0.95 | <0.001 | 0.97 | 0.94, 0.99 | 0.018 |

**Supplementary Table 3. Univariate and multivariate analysis of variables associated with overall survival.** AI = autoimmune, ASPS = alveolar soft part sarcoma, concomitant TTT = concomitant treatment, 95% CI = 95% confidence interval, CRP = C-reactive protein, DDLS = dedifferentiated liposarcoma, DNLR = derived neutrophil lymphocyte ratio, ECOG PS = Eastern Cooperative Oncology Group Performance Status, FNCLCC = fédération nationale des centres de lutte contre le cancer, GIST = gastro-intestinal stromal tumor, HR = hazard ratio, ICI = immune checkpoint inhibitor, LDH = lactate dehydrogenase, LMS = leiomyosarcoma, TLS = tertiary lymphoid structure, TMB = tumor mutational burden.

| **Characteristic** | **Univariate analysis** | | | | **Adjusted multivariate analysis** | | |
| --- | --- | --- | --- | --- | --- | --- | --- |
|  | **N** | **OR***^1^* | **95% CI** | **p-value** | **OR***^1^* | **95% CI** | **p-value** |
| **Age at initiation** | 271 | 0.99 | 0.98, 1.01 | 0.4 |  |  |  |
| **Sex** | 272 |  |  | 0.2 |  |  |  |
| Female |  | — | — |  |  |  |  |
| Male |  | 1.38 | 0.87, 2.21 |  |  |  |  |
| **ECOG PS** | 272 |  |  | 0.007 |  |  |  |
| 0 |  | — | — |  |  |  |  |
| 1 |  | 0.48 | 0.30, 0.76 |  |  |  |  |
| 2+ |  | 0.50 | 0.12, 1.37 |  |  |  |  |
| **Location** | 272 | 1.50 | 0.58, 3.20 | 0.4 |  |  |  |
| Abdomen/pelvis | 271 |  |  | 0.3 |  |  |  |
| Limb |  | — | — |  |  |  |  |
| Head/neck |  | 1.00 | 0.58, 1.68 |  |  |  |  |
| Thorax |  | 1.37 | 0.52, 3.01 |  |  |  |  |
| **Histotype** |  | 0.53 | 0.22, 1.11 |  |  |  |  |
| Other | 272 |  |  | <0.001 |  |  | <0.001 |
| ASPS |  | — | — |  | 1.00 | — |  |
| Chordoma |  | 8.82 | 3.57, 21.1 |  | 3.99 | 1.46, 10.8 |  |
| GIST |  | 3.19 | 1.60, 5.99 |  | 3.05 | 1.35, 6.60 |  |
| LMS |  | 2.14 | 1.06, 4.05 |  | 1.11 | 0.49, 2.46 |  |
| DDLPS |  | 0.54 | 0.20, 1.19 |  | 0.51 | 0.18, 1.26 |  |
| **Genomic profile** |  | 0.23 | 0.01, 1.08 |  | 0.24 | 0.01, 1.17 |  |
| Complex | 272 |  |  | 0.001 |  |  | 0.2 |
| Simple |  | — | — |  | 1.00 | — |  |
| **FNCLCC grade** |  | 2.12 | 1.34, 3.34 |  | 1.66 | 0.82, 3.34 |  |
| 1 | 157 |  |  | 0.2 |  |  |  |
| 2 |  | — | — |  |  |  |  |
| 3 |  | 4.82 | 0.96, 87.6 |  |  |  |  |
| **Stage at diagnosis** |  | 3.40 | 0.69, 61.4 |  |  |  |  |
| Localized or locally advanced | 272 |  |  | 0.2 |  |  |  |
| Metastatic |  | — | — |  |  |  |  |
| **Stage at initiation** |  | 1.35 | 0.84, 2.13 |  |  |  |  |
| Locoregional or locally advanced | 272 |  |  | 0.050 |  |  |  |
| Metastatic |  | — | — |  |  |  |  |
| **NF1 mutation** |  | 0.44 | 0.22, 1.00 |  |  |  |  |
| **PDL1 expression** | 109 | 1.58 | 0.37, 4.54 | 0.5 |  |  |  |
| **P53 mutation** | 99 | 0.00 |  | 0.4 |  |  |  |
| **RB1 mutation** | 120 | 0.54 | 0.22, 1.21 | 0.14 |  |  |  |
| **DNMT3 mutation** | 115 | 0.16 | 0.01, 0.75 | 0.015 |  |  |  |
| **PIK3CA mutation** | 105 | 3.02 | 0.87, 8.08 | 0.078 |  |  |  |
| **TERT mutation** | 114 | 5.91 | 1.62, 17.4 | 0.010 |  |  |  |
| **AMT mutation** | 109 | 3.74 | 0.58, 13.8 | 0.14 |  |  |  |
| **ATRX mutation** | 108 | 1.68 | 0.27, 5.72 | 0.5 |  |  |  |
| **CDKN2A mut** | 106 | 1.00 | 0.24, 2.85 | >0.9 |  |  |  |
| **CDKN2B mut** | 118 | 1.29 | 0.38, 3.34 | 0.6 |  |  |  |
| **PTEN mutation** | 108 | 0.79 | 0.13, 2.65 | 0.7 |  |  |  |
| **Presence of TLS** | 117 | 0.33 | 0.02, 1.56 | 0.2 |  |  |  |
| **TMB (mut/Mb)** | 42 | 0.49 | 0.16, 1.42 | 0.2 |  |  |  |
| **Metastatic sites** | 98 | 1.02 | 0.93, 1.09 | 0.6 |  |  |  |
| 0 | 272 |  |  | 0.042 |  |  |  |
| 1 |  | — | — |  |  |  |  |
| 2 |  | 0.76 | 0.35, 1.92 |  |  |  |  |
| 3 and more |  | 0.54 | 0.25, 1.37 |  |  |  |  |
| **Lung** |  | 0.35 | 0.15, 0.93 |  |  |  |  |
| **Liver** | 272 | 0.68 | 0.43, 1.07 | 0.094 |  |  |  |
| **Peritoneum** | 272 | 1.05 | 0.62, 1.71 | 0.9 |  |  |  |
| **Brain** | 272 | 0.85 | 0.50, 1.40 | 0.5 |  |  |  |
| **Bone** | 272 | 1.42 | 0.43, 3.47 | 0.5 |  |  |  |
| **Soft tissue** | 272 | 0.50 | 0.26, 0.90 | 0.019 |  |  |  |
| **Adrenal gland** | 272 | 0.48 | 0.25, 0.86 | 0.012 |  |  |  |
| **Pancreas** | 272 | 0.55 | 0.09, 1.76 | 0.4 |  |  |  |
| **Lymph node** | 272 | 0.61 | 0.10, 1.95 | 0.5 | 0.58 | 0.30, 1.04 | 0.066 |
| **Other** | 272 | 0.54 | 0.30, 0.93 | 0.026 |  |  |  |
| **Number of prior**  **systemic lines** | 272 | 0.88 | 0.31, 1.99 | 0.8 |  |  |  |
| 0 |  | — | — |  |  |  |  |
| 1 |  | 0.65 | 0.33, 1.34 |  |  |  |  |
| 2 |  | 0.39 | 0.18, 0.85 |  |  |  |  |
| 3plus |  | 0.35 | 0.17, 0.74 |  |  |  |  |
| **ICI type** | 272 |  |  | 0.4 |  |  |  |
| Anti-PD1 |  | — | — |  |  |  |  |
| Anti-PDL1 |  | 0.81 | 0.51, 1.28 |  |  |  |  |
| **Associated ICI** | 272 |  |  | 0.8 |  |  |  |
| None |  | — | — |  |  |  |  |
| Anti-CTLA4 |  | 1.30 | 0.60, 2.49 |  |  |  |  |
| Anti-LAG3 |  | 1.14 | 0.28, 3.10 |  |  |  |  |
| **Concomitant TTT** | 272 |  |  | 0.004 |  |  |  |
| None |  | — | — |  |  |  |  |
| Antiangiogenic |  | 1.11 | 0.58, 1.99 |  |  |  |  |
| Anti-MEK |  | 0.34 | 0.14, 0.73 |  |  |  |  |
| Other |  | 0.76 | 0.18, 2.12 |  |  |  |  |
| Chemotherapy |  | 0.31 | 0.12, 0.69 |  |  |  |  |
| Radiotherapy |  | 0.50 | 0.22, 1.03 |  |  |  |  |
| **DNLR** | 269 | 0.78 | 0.64, 0.94 | 0.006 | 0.78 | 0.61, 0.97 | 0.022 |
| **LDH (UI/L)** | 216 | 1.00 | 1.00, 1.00 | 0.061 |  |  |  |
| **CRP (mg/L)** | 114 | 0.99 | 0.97, 1.00 | 0.003 |  |  |  |
| **Albumin (g/L)** | 257 | 1.05 | 1.01, 1.09 | 0.016 |  |  |  |

**Supplementary Table 4. Univariate and multivariate analysis of variables associated with progression-free survival superior to 6 months**. AI = autoimmune, ASPS = alveolar soft part sarcoma, concomitant TTT = concomitant treatment, 95% CI = 95% confidence interval, CRP = C-reactive protein, DDLS = dedifferentiated liposarcoma, DNLR = derived neutrophil lymphocyte ratio, ECOG PS = Eastern Cooperative Oncology Group Performance Status, FNCLCC = fédération nationale des centres de lutte contre le cancer, GIST = gastro-intestinal stromal tumor, HR = hazard ratio, ICI = immune checkpoint inhibitor, LDH = lactate dehydrogenase, LMS = leiomyosarcoma, TLS = tertiary lymphoid structure, TMB = tumor mutational burden.

| **Abnormality** | **Univariate analysis** | | | | **Adjusted multivariate analysis** | | |
| --- | --- | --- | --- | --- | --- | --- | --- |
|  | **N** | **HR** | **95% CI** | **p-value** | **HR** | **95% CI** | **p-value** |
| **nf1** | 91 | 0.52 | 0.22, 1.24 | 0.11 | 0.13 | 0.03, 0.53 | **<0.001** |
| **PDL1/CD274** | 87 | 3.78 | 0.51, 28.2 | 0.3 |  |  |  |
| **p53** | 91 | 1.26 | 0.81, 1.96 | 0.3 |  |  |  |
| **rb1** | 91 | 2.41 | 1.38, 4.20 | 0.004 | 2.08 | 1.12, 3.84 | **0.025** |
| **dnmt3a** | 91 | 0.51 | 0.22, 1.18 | 0.085 |  |  |  |
| **pik3ca** | 91 | 0.91 | 0.29, 2.91 | 0.9 |  |  |  |
| **tert** | 91 | 1.00 | 0.25, 4.10 | >0.9 |  |  |  |
| **atm** | 91 | 1.09 | 0.34, 3.47 | 0.9 |  |  |  |
| **atrx** | 91 | 1.07 | 0.54, 2.15 | 0.8 |  |  |  |
| **cdkn2a** | 91 | 0.95 | 0.46, 1.98 | 0.9 |  |  |  |
| **cdkn2b** | 91 | 0.95 | 0.46, 1.98 | 0.9 |  |  |  |
| **pten** | 91 | 0.84 | 0.40, 1.75 | 0.6 |  |  |  |
| **Presence of TLS** | 15 | 1.16 | 0.34, 3.98 | 0.8 |  |  |  |
| **TMB (mutation/Mb)** | 91 |  |  | 0.10 |  |  |  |
| < 10 |  | 1.00 | — |  |  |  |  |
| $\geq$10 |  | 0.36 | 0.09, 1.50 |  |  |  |  |

**Supplementary Table 5. Subgroup analysis of molecular alterations associated with progression-free survival (n=91)**. 95% CI = 95% confidence interval, HR = hazard ratio, TLS = tertiary lymphoid structure, TMB = tumor mutational burden.

**Supplementary Figure 3. Kaplan-Meier curves of progression-free survival according to NF1 and RB1 mutational status.** Tick marks indicate censoring of data. HR = hazard ratio, mut = mutation, wt = wild type, mPFS = median progression-free survival, 95%CI = 95% confidence interval.


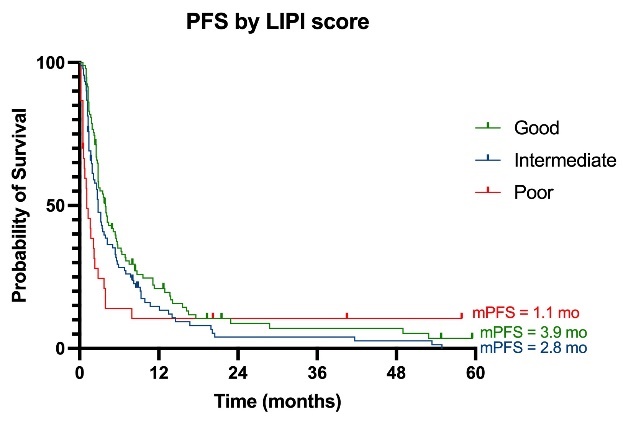

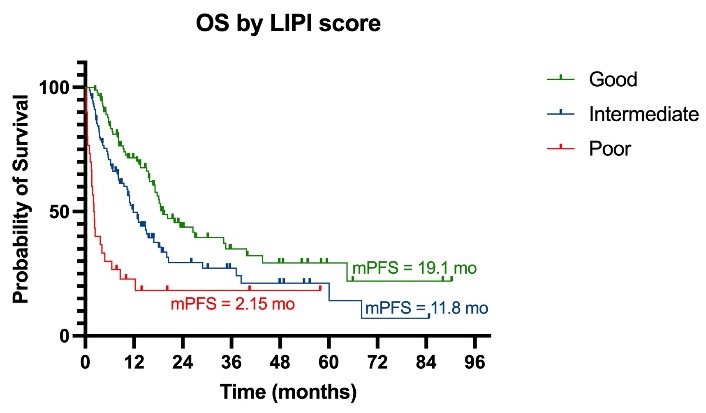


**Supplementary Figure 4. Kaplan-Meier curves of progression-free survival and overall survival according to LIPI score.** Tick marks indicate censoring of data. HR = hazard ratio, OS = overall survival, PFS = progression-free survival, mOS = median overall survival, mPFS = median progression-free survival, 95%CI = 95% confidence interval.

| Safety | N = 272 |
| --- | --- |
| Immunotherapy related adverse events (irAEs) (at least grade 1) – n (%) | 151 (56%) |
| Dermatologic | 66 (24%) |
| Grade 3-4 | 1 (<1%) |
| Endocrine | 56 (21%) |
| Grade 3-4 | 2 (<1%) |
| Rhumatologic | 12 (4.4%) |
| Grade 3-4 | 0 (0%) |
| Digestive | 44 (16%) |
| Grade 3-4 | 3 (1%) |
| Hepatic | 26 (9.6%) |
| Grade 3-4 | 0 (0%) |
| Muscular | 17 (6.2%) |
| Grade 3-4 | 3 (1%) |
| Respiratory | 8 (2.9%) |
| Grade 3-4 | 0 (0%) |
| Cardiac | 7 (3%) |
| Grade 3-4 | 3 (1%) |
| Nephrologic | 2 (<1%) |
| Grade 3-4 | 0 (0%) |
| Other (sarcoidosis, vascularitis, infusion related reaction, lymphocytary sialadenitis) | 7 (3%) |
| Grade 3-4 | 1 (<1%) |

**Supplementary Table 6. Safety data**
